# Placebo and the law of identification

**DOI:** 10.3389/fpsyt.2024.1474558

**Published:** 2024-12-06

**Authors:** Steve F. Bierman, Andrew Weil, Stephen Dahmer

**Affiliations:** College of Medicine, University of Arizona, Tucson, AZ, United States

**Keywords:** placebo, nocebo, placebo effect, nocebo effect, placebo response, nocebo response

## Abstract

Thousands of essays and studies have been published on placebo and nocebo. Yet, despite this plethora of information, we are not much closer to a comprehensive understanding of the fundamental mechanism producing placebo and nocebo effects than we were in 1946, when participants in the Cornell Conferences on Therapy speculated on the roles of authority, belief and expectancy. In this paper, we examine the weaknesses in current placebo and nocebo definitions and theories. We also propose a more concise and comprehensive definition and theory of placebo and nocebo by introducing the Law of Identification and the Generic Placebo Instruction (GPI). The latter being the placebo/nocebo information expressed or implied in virtually every clinical encounter and trial; the former (i.e., the Law of Identification), being what drives the GPI to actualization. Further, we demonstrate the explanatory power of this new theory and suggest clinical studies that test predictions arising from it - studies whose results, if positive, would translate universally into clinical practice.

## Introduction

Over the last century, thousands of essays and studies have been published on placebo and nocebo (1–4). Yet, despite this plethora of information, we are not much closer to a comprehensive understanding of the fundamental mechanism producing placebo and nocebo effects than we were in 1946, when participants in the Cornell Conferences on Therapy speculated on the roles of authority, belief and expectancy (5). True, other factors have been added to the discussion. For example, patient factors - like attitude, mindset and meaning (6); caregiver factors - like time spent with the patient and caregiver demeanor; and environmental factors - like the milieu in which therapeutic measures are offered (7, 8). Attention has even been directed to the patient-caregiver relationship (9). But, no unifying theory for this welter of observations has emerged. On the contrary, unanswered questions abound: What are the essential elements of placebo and do they differ from those of nocebo? Are placebo and nocebo effects distinct from each other and produced through different mechanisms, or is there a common mechanism or law that subsumes both?

The purpose of this paper is to (a) examine the weaknesses in current placebo and nocebo definitions and theories, (b) to propose a more concise and comprehensive definition and theory by introducing the Law of Identification and the Generic Placebo Instruction (c) to demonstrate the explanatory power of this new theory, and (d) to suggest future studies that test predictions arising from this new theory - studies whose results, if positive, would translate universally into clinical practice.

## History of the placebo concept

A brief history of placebo concepts, as reflected in the evolution of their definitions, will serve to highlight our current predicament.

Etymologists trace the origins of the word *placebo* to the word *Ethalekh* in the Hebrew bible, which was translated into Latin using the root *placere* meaning “to please, give pleasure, be approved, be pleasing, be agreeable, acceptable, to suit, satisfy.” (10)

It was not until the late 18^th^ century that medical dictionaries, like Motherby’s *New Medical Dictionary (1785)*, began to include the word *placebo:* defined as “a commonplace [pejorative connotation] method or medicine” used to placate patients’ expectations of treatment. Thus, placebo was originally conceived and used in medicine as a means of pleasing patients with such curatives as bread pills, colored water and other nostrums.

Gradually, as clinical interests and experimental methods evolved, so too did the definition of placebo. *Webster’s New International Dictionary (1933)* was the first to include the word “inactive” in the definition: “a medicine, or preparation, especially an inactive one, given merely to satisfy a patient.” In 1949, Blakiston’s *New Gould Medical Dictionary* limited the definition of placebo to “inactive medicines.” And in 1951, Dorland’s *American Illustrated Medical Dictionary* defined placebo as “an inactive substance or preparation formerly given to please or gratify a patient, now also used in controlled studies to determine the efficacy of medicinal substances.”

It was also in the mid-20^th^ century, at the Cornell Conferences on Therapy, that the phrase “placebo effect” – namely, the consequence of using such “dummy” medicines - came into usage (5). In the same period, the terms “toxic or negative placebo effects” (later, called nocebo effects) were popularized (3, 10). In 1960, the third edition of the *Psychiatric Dictionary* dispensed with the purpose of “humoring” patients and acknowledged psychological and psychophysiologic effects, inactive and active agents, and the role of placebo as an effective control in research. Moreover, this definition proposed a mechanism by which placebo works: namely, through the patient’s expectations and other psychological factors.

By 1961, the conceptual framework around placebo phenomena had evolved to the point where Shapiro was able to define placebo as “a therapeutic procedure (or that component of any therapeutic procedure) which is given deliberately to have an effect, or unknowingly has an effect on a patient, symptom, syndrome or disease, but which is objectively without *specific* activity for the condition being treated.” (10) Additionally, he described placebo’s usefulness as a control for research and distinguished between placebo effect and placebo *per se*, defining the former as “the changes produced by placebos.” (ibid, p.1225)

In 1982, Brody defined the placebo effect as “the change in the patient’s condition that is attributable to the symbolic import of the healing intervention rather than to the intervention’s specific pharmacologic or physiologic effects.” (9) And, he defined placebo as: “a form of medical therapy, or an intervention designed to simulate a medical therapy, that is believed to be without specific activity for the condition being treated, and that is used either for its symbolic effect or to eliminate observer bias in a controlled experiment.”

In 2013, Kirsh drew a useful distinction – hinted earlier by Benson (8) and others - between placebo effect and placebo response by explaining that in the absence of a “natural history control” a randomized placebo-controlled trial (RCT) could not discern events caused by placebo versus events consequent to the natural history of the condition under study. Hence, he argued, the term “*placebo response*” should be used when referring to results from a two-arm placebo-controlled study; whereas, “placebo effect” should be reserved for placebo-induced results not attributable to the natural history, as determined in a three-arm RCT (i.e., placebo, natural history and experimental groups) (11). Blease and colleagues extended this distinction by specifying that placebo effects are “changes attributable … to psychobiological mechanisms associated with the therapeutic encounter.” (12)

Finally, an Oxford University Press (2023) compendium on placebo offered the following:

“*Placebo effects* refer to a beneficial effect produced by a placebo drug or treatment or a manipulation of the participants belief, which cannot be attributed to the properties of the placebo/manipulation itself and is, therefore, due to the cascade of neurobiological changes related to expectancy, prior therapeutic experiences, observation of benefit in others, contextual and treatment cues, and interpersonal interactions.” (13)

Three concepts emerge from these various definitions – namely, (a) *placebo* (and *nocebo*), (b) *placebo* (and *nocebo*) *effects*, and (c) the *mechanism(s)* driving placebo and nocebo effects. Placebo has evolved conceptually from the act of pleasing patients with dummy remedies to “drugs, treatments or manipulations of the participants beliefs or expectancies” that “… lack the potential to produce benefit [or harm] on the basis of pharmacological properties or physical manipulations.” (13). Placebo or nocebo effects are currently viewed as a broad array of beneficial or deleterious clinical and laboratory outcomes resulting from the administration of placebos or nocebos. The mechanisms causing such effects are attributed to beliefs or expectancies, conditioning, observational learnings, context and cures, and/or interpersonal interactions – all presumably intermediated by neurobiological sequences (13).

As we shall see, these current understandings – while having spawned much fruitful research – suffer from what David Deutsch calls limited *reach* (meaning, they do not encompass much of the observed phenomena) and excessive *variability* or vagueness in their terms (14). In other words, despite their extended evolution, the current understandings of placebo, placebo effects and their underlying mechanisms are imperfect scientific explanations. This should come as no surprise, for as Deutsch reminds us, “…original sources of scientific theories are almost never good sources. How could they be? All subsequent expositions are intended to be improvements on them, and … improvements are cumulative.” (15) Which is to say, no precise scientific explanation evolves but for its imperfect antecedents.

## The problems with current placebo/nocebo concepts

### Placebo/nocebo

Let’s explore what is imperfect about the concept of placebo/nocebo as “an inert drug or treatment,” or a “manipulation of the patient’s or participant’s beliefs.”

The first part of this definition suggests that it is the material thing or deed *per se* that causes placebo/nocebo effects. And yet, placebos consist of a vast array of pills, sprays, phony inhalations, sham surgeries, inactive creams, fake procedures, inert infusions and so on. Is it even credible to think that, despite their gaping differences, these nostrums and sleights, by virtue of their material characteristics alone, somehow evoke healing responses whenever they are deployed? Do sugar pills and saline injections - which can both relieve pain – stimulate some hypothetical placebo-receptor that then triggers the intermediary neurobiological steps leading to pain relief? If so, they must always activate that receptor regardless whether a clinical trial is being conducted or not – which is absurd. Moreover, if there were a common material pathway, aren’t we then left to assume that all other varieties of placebo that alleviate pain must also activate that same pathway? And further, that this universal route also actuates the various other causal sequences that account not only for pain relief but for all other placebo effects as well. Of course, there is no evidence for this common material pathway. And even if there were, how could it possibly explain the fact that placebo effects are also elicited by non-material means: for example, “the manipulation of the patient’s or participant’s belief”?

Moreover, the notion of a universal material pathway leading from placebo to placebo effects leaves open the question: How does the material thing called placebo also account for noxious or nocebo effects – often, in one and the same person? The answer: It does not.

Finally, as one researcher observed, to claim that these so-called inert substances or procedures themselves produce beneficial (and deleterious) outcomes is “unhelpful as well as illogical.” (13, p240) Either the thing is inert, or it is active; it cannot be both. The fake cream, for example, cannot be both inert with respect to a patient’s rash and active against it. A sham surgery either addresses a patient’s defective part, or it does not. In other words, by virtue of the very fact that it is inert, the material “prop” we call placebo cannot be the true cause of the placebo effect.

Hence, placebo is neither a thing nor a deed.

What then of placebo/nocebo as a “manipulation of the patient’s or participant’s belief”? Here we should understand that the placebo is considered to be the manipulation. Later, when discussing mechanisms, we will take up its purported effect on belief.

So, what precisely is meant by this term “manipulation”?

In trials and experiments wherein a material placebo is absent, the manipulation sometimes takes the form of a written instruction (16); in other instances, it is a verbal instruction (17); in yet others, it is an implied message (18); and, at times, it appears the manipulation is conveyed via body language or other non-verbal cues (19). In the first instance (written instruction), the manipulation comes in the form of a pamphlet offering information about the genetic determinants of exercise tolerance. In the second (verbal instruction), participants are told of the possible effects of the intervention on their back pain. In the third instance (implied message), a subset of participants is simply told what drug they are being given – thereby implying the possibility of the drug’s well-known side effects. In the fourth (non-verbal message), clinicians were informed they would not be administering fentanyl, despite the fact that they were; nothing was ostensibly said, written or implied to the patient. Yet, fentanyl failed to provide pain relief in patients whose doctors believed they were not administering an analgesic. There are scores of other similar manipulations throughout the placebo literature.

Are we to assume that each and every one of these placebo/nocebo manipulations is unique to its specific setting – that there is no common element, no unifying principle? Or is there, as Beecher first speculated in 1955, a common element to them all? (3) The current definition would suggest there is not. In fact, all we can gather from the current conception is that placebo-manipulations somehow involve the communication of information through one of a variety of channels to the patient or participant. So far as we know, that information can be virtually anything that affects the receiver’s beliefs - the possibilities are myriad.

This is what David Deutsch means by *variability.* As mentioned above, *variability* is a common feature of imperfect explanations (14). A better explanation would specify the common structure or substructure of the information inducing the placebo/nocebo effect – provided it can be shown to exist.

But *variability* is not the only flaw with this conception of placebo; there are other problems. Notice, nothing is specified regarding the source of the information or the nature of the patient’s or participant’s relationship to that source. And yet, perhaps most importantly as it relates to clinical care, the evidence strongly suggests that both elements play a role in placebo/nocebo responses (13). Moreover, the present definition is silent on the qualities and circumstances of the patients or participants, as if any given manipulation will have one and the same effect on each and every individual, regardless of their differences.

Let’s look more closely at these issues, beginning with the source communicating the information. In doing so, bear in mind we are presently discussing what constitutes the actual placebo – that is, the intervention. So far, we have established that placebo not a material intervention *per se*; rather, it is a noetic (meaning, of or relating to mind) intervention of a sort presently unspecified. It is true that the source of the intervention and its relationship to the placebo have been studied considerably by scientists in the field. However, those efforts have been directed, by and large, toward trying to understand the *mechanisms* that mediate placebo effects. As we shall see, this mistaken approach has contributed to the current state of “ambiguous and/or insufficient theoretical orientations,” and so, placebo and placebo effects have remained “somewhat enigmatic to both research and medical practices.” (ibid,p253)

Early placebo researchers concluded, “There can be no doubt that the placebo derives its power from the … relationship between the omnipotent physician and the needs of the patient.” (20) Further, they observed that with respect to anti-anxiety treatment, “…the proportional response to optimistic doctors was more than double that to the pessimistic doctors…,” (21) and that “the demeanor of the physician” is a pertinent factor (8). More recently, researchers have established that placebo effects are enhanced when clinicians or scientists demonstrate “competence and empathy.” (22, 23) In other words, the evidence suggests that the source of the placebo-manipulation cannot be just anyone; rather, the person or persons delivering the information must meet certain determinative criteria. Clearly, a diffident vagabond in ragged garb would not induce the same placebo effect - delivering the same information, to the same patient – as a confident and caring, white-coated physician.

As to the relationship between source and subject, numerous observations attest to its significance. Early on, Brody noted that drugs administered by a physician confident in their outcome performed superior to placebo; but, he observed, those same drugs “showed no difference [from placebo] when administered by a less supportive and more skeptical physician.” (6) Along those lines, Zilcha-Mano and colleagues have demonstrated that the therapeutic alliance “…can predict symptomatic change in psychopharmacology…” for both the treatment and the placebo groups (24). Others have found that trust between patient and practitioner is “a critical element.” (25) And a “dose-response” enhancement of the placebo effect has been demonstrated when empathy, thoughtful listening and targeted symptom inquiry are aspects of the relationship (26). In sum, there is mounting evidence to indicate that “a warm, trusting, and positive patient-provider encounter can enhance placebo effects.” (13) Therefore, any explanation of placebo must somehow take into account the relationship between source and subject and, ideally, discern common determinative elements within what now appear to be the many disparate qualities of that relationship.

Lastly, let’s explore the characteristics of the patient or participant.

Presently, one cannot predict who within a given placebo cohort will respond to placebo and who will not (9, 13). Even if one could make such a prediction, so-called placebo responders tend not to be stable over time (3). In other words, the determinants of placebo-responsivity appear to be somehow contingent on varying characteristics within the patient/participant. But what are those characteristics?

Beecher “…found strong evidence that placebos are far more effective in relieving a stressful situation … when the stress is severe, than when it is less so.” (3) Shortly thereafter, Benson cited multiple studies indicating, “The higher the level of patient concern and the greater the discomfort, the more likely relief from a placebo will occur.” (8) Similarly, Hauser noted, regarding nocebo, that patients “…are highly receptive to negative suggestion, particularly in situations perceived as existentially threatening, such as impending surgery, acute severe illness, or an accident.” (27) Horing and colleagues performed an extensive systematic review looking for indicators of placebo responsivity and found, “that predictors are rather not found among ‘classic’ trait personality variables….” (28) However, they did discover that an “… important cluster seems to revolve around an internality-externality dimension, such that participants follow placebo instructions more readily when having an external locus of control.”(ibid) In other words, evidence suggests that a patient/participant who is free of stress or discomfort, and who is fully self-reliant and in need of no succor, is less likely to respond to a placebo than a stressed and needy patient in quest of external assistance.

Summing up, we acknowledge that placebo is not and cannot be a material intervention. Rather, it is a noetic intervention – a communication (implicit or explicit) of information to an individual with specific characteristics, by a person also with specific characteristics, via a relationship, again, of a specified nature. To avoid the explanatory defects Deutsch cautions against, it behooves us to identify precisely those specific characteristics that define the subject, source, and relationship – as well as the information being conveyed.

### Placebo effects

Let us now consider *placebo effects* and, thereafter, the currently favored explanations of the *mechanisms* which bring about those effects.

In 2013, as previously mentioned, Kirsch noted that “the placebo effect is the difference between the placebo response and the changes that would be observed even without the administration of a placebo. To assess the placebo effect, one has to subtract changes due to the natural history of the disorder….” (11) Accordingly, we should note that many of the purported placebo “effects” in the literature are not observant of this distinction; by and large, they issue from two-arm studies, rather than three-arm studies with a natural history control group.

Nevertheless, it is worth briefly outlining the reported range of placebo responses and effects. We begin by observing, as Beecher did, that “it must not be supposed that the action of placebos is limited to ‘psychological’ responses.” (3) In fact, placebo effects are elicited in virtually “all medical [and surgical] procedures.” (8) For example, subjective or psychological placebo responses include: acute and chronic pain reduction (29–31), appetite improvement in cancer patients (32), anti-anxiety and anti-depressant effects, and more (13). Sham surgeries have induced favorable responses in angina pectoris (33, 34), knee osteoarthritis (35), sleep apnea (36), obesity (37) and more (38). And placebo medicaments have been shown to cause hair growth (39) and hair loss (40); to induce measurable improvements in juvenile rheumatoid arthritis (41), diabetic neuropathy and post-herpetic neuralgia (42), hypertension (43), coronary artery revascularization (44), food sensitivity (45), “laboratory values and other measures of physiologic change,” diabetes and malignant neoplasms (6) and a broad array of other medical conditions (13). In fact, even when the factor of deception is eliminated by using an open-label approach, placebo responses have included relief from pain (31), allergic rhinitis, cancer-related fatigue, menopausal hot flashes, attention-deficit/hyperactivity disorder, depression and irritable bowel syndrome (46). Rare, indeed, is the malady that cannot be aided by placebo (47).

In other words, the range of placebo responses/effects encompasses a broad spectrum of both psychological and physical changes – changes that led Norman Cousins in 1988 to conclude: “… the physician has a prime resource at his disposal in the form of the patient’s own apothecary.” (48)

However, we should not confine our survey to purely salutary responses. Noxious or nocebo responses, which often occur in the very same individual that is experiencing a positive placebo response, include: vomiting, nausea, headache (2); dry mouth, difficulty concentrating, drowsiness, relaxation, fatigue, sleep, palpitations, itching, rash, epigastric pain, diarrhea, urticaria, angioneurotic edema (3); altered complete blood count, alopecia (40); fever, myalgia, coryza, soreness at injection site (49); vision problems, constipation, anorexia, sexual dysfunction (27); shortness of breath, acute pain (50); and a host of other psychological and physiological symptoms (13). In other words, virtually every negative symptom – psychological or physiological - that can be evoked by an active medication or manipulation can also be evoked, in some measure, by a placebo/nocebo intervention.

In sum, we observe that placebo/nocebo interventions somehow elicit a wide array of psychological and physiological placebo/nocebo responses and effects. Some of these responses – hair growth and hair loss, for example – likely entail the expression or repression of the patients’ or participants’ genes. Others somehow involve the operation of neurobiologic intermediaries, like endorphins, endocannabinoids or neurotransmitters (13). Still others operate by yet-undiscovered means.

The question is: How does placebo/nocebo, which we now understand to be a noetic intervention – namely, the communication of information – effectuate such a vast range of positive and negative outcomes? Before proposing an explanation that meets Deutsch’s requirements, let’s examine some of today’s most prevalent explanations – explanations that led one philosopher of science to comment, “the medical and psychiatric literature on placebos and their effects is conceptually bewildering, to the point of being a veritable Tower of Babel.” (51)

### Mechanisms

#### Expectancy

Currently, the most favored explanation for the production of placebo/nocebo effects is the *expectancy theory.* Despite the fact that some authors maintain “there is no consensus on exactly how to define *expectations”* (13), one expert consensus asserts that placebo/nocebo effects “…occur in clinical or laboratory medical contexts, respectively, after administration of an inert treatment or as part of active treatments, due to mechanisms such as expectancies of the patient.” (52) This consensus further defines expectation as, “Constructs that refer to anticipation of outcome that are verbalized and measurable via validated scales;” and they define expectancy as: “A psychophysical predictor that can be present in humans and non-humans without full awareness (implicit expectancies).”(ibid.) In other words, we are left with: an expectation or expectancy is a conscious or unconscious mental event that entails the anticipation of some outcome, and that due to unspecified “mechanisms” produces placebo/nocebo effects.

Is this an explanation? Even if we were to grant that expectation and expectancy are well-defined terms, are we any closer to understanding how they produce placebo/nocebo effects? What causes an expectation or expectancy to initiate the series of neurobiological events within the subject that intermediate the final placebo response? (13) We don’t know and, unfortunately, cannot know from this theory.

But the terms of expectancy theory are not sharply defined, which allows for statements like this: “A doctor, nurse, or psychotherapist can rather easily convey negative expectations to patients.” (49) Here we descry one of the major shortcomings of the theory. No person conveys expectations to another person. Rather, expectations are secondary effects within the recipient of a communication. For example, imagine you are told that astronomical authorities predict a solar eclipse tomorrow. If you credit the communicator and thereby believe the experts’ predictions, then and only then do you come to expect an eclipse. In other words, expectations arise subsequent to (a) the receipt of an idea from a creditable source and (b) assessing the probability of its future actualization. Expectancy is always derivative; it is always secondary to crediting the initial idea and anticipating its future actualization.

So, why build a theory around an event several steps into the causal sequence under study? It is tantamount to saying, the ball rolls down the incline plane due to its momentum, ignoring entirely the Law of Gravity.

Kelley astutely observes: “The causal chain begins when information … is provided to the patient.” (50) Wouldn’t it be more fruitful, then, to examine the information that initiates the sequence? This is exactly what Silvestri and colleagues did, for example, when they ascribed “patient knowledge” as the initial cause of their discovery that erectile dysfunction (ED) in males on beta-blockers is directly proportional to the amount of information they know about ED as a possible side effect (18). In other words, the causal sequences that eventuate in both nocebo and placebo effects begin with information – not expectation, which may or may not occur, and is always secondary.

But inexact definitions and misattributed secondary effects (i.e. expectation/expectancy) are not the only issues we discover with expectancy theory. What of the notion of unconscious expectations? True, most of the work on expectation theory involves conscious expectation; in fact, the validated tools for measuring such expectation rely almost entirely on conscious reporting (53). Yet, unconscious expectation is often inferred when so-called placebo effects result from interventions that generate no obvious conscious activity. More specifically, this terminology is often used by expectancy theorists to account for conditioned responses. For example, Mommaerts writes that following the pairing of a morphine injection with the contextual cues of the experimental chamber, Pavlov’s dogs became sedated prior to actual re-injection owing to “their expectation of being re-injected.” (54) He goes on to state, despite Pavlov having refrained from such constructions, that “Pavlov’s dogs expected food when the bell tolled….” Many other authors have extended this idea of unconscious expectation to so-called placebo and nocebo responses in humans (13, 55).

We should beware this conflation of conditioned responses with placebo responses, for it requires an unnecessary and untestable notion – namely, unconscious expectation. Two events are temporally paired, and a pattern is thereby formed; regardless what conscious or unconscious effects this pattern may elicit, it will have a tendency to repeat for the simple reason that patterns persist; or, if you prefer the more modern formulation, “neurons that fire together, wire together.” (56) This is the Law of Association. As we shall see, it is entirely different from the Law of Identification which governs placebo/nocebo effects (57). Therefore, we agree with Benedetti and colleagues who assert, “… conscious expectation and unconscious conditioning are involved in different circumstances…,” and so, “… the differentiation between expectation [meaning, in this instance, placebo phenomena] and conditioning is important.” (58)

Beyond its untestable constructs, we find yet other problems with the expectancy theory. Consider, for example, a study that claims to be “the first prospective, causal evidence for patient expectancy as a mediator of placebo effects in antidepressant clinical trials.” (59) This study does what all placebo-controlled studies should be required to do – namely, quote exactly the instructions given to each group in the study, and describe in detail the metrics by which the variables are assessed. Rutherford and colleagues told depressed patients, who were 100% guaranteed to receive the anti-depressant drug, that the agent “… has been proven effective for the treatment of depression in patients like you.”(ibid.) Whereas, patients who were randomly assigned to a group that would receive either placebo or citalopram were told: “There is a chance you will receive the antidepressant … proven effective for the treatment of depression in patients like you. There is also a chance you will receive placebo … not specifically effective for depression.” After receiving this information, expectation of “being completely better” or “better” at the end of the study was assessed. (Note: expectation of feeling “no better” or “worse” was not assessed.) The group with 100% certainty of receiving the drug experienced (on average) higher expectancy of improvement and (again, on average) higher response rates (53.8%) than the placebo-control group (25%).

At first sight, this appears to be compelling evidence in support of the expectancy theory. However, rather than attributing expectancy as the cause of the observed outcomes, one might just as deftly explain the results as a consequence of the nocebo effect. After all, the placebo-controlled group received a negative suggestion – namely, you may receive an agent “not specifically effective for depression.” It is entirely conceivable that this idea became operative within the placebo-controlled cohort, directly causing fewer therapeutic responses.

Notice, too, that this study is not evidence of a “causal” connection between expectancy and placebo effect. Rather, it is evidence of a correlation between the average expectancy score in a group and the average number of therapeutic responses in that same group. Therefore, it tells us nothing about the effects of expectancy at the individual level. What if within the 100% certainty group there was a patient in whom expectancy was high and responsivity low? That one anomaly should cast serious doubt over the expectancy theory, and cause us to strive for refinements or revisions. Similarly, what if a patient among the placebo responders had low expectations and high responsivity? That, too, should drive a disconfirming cloud over the theory. Unfortunately, those patient-specific data were not made available in this trial; nor are they available in most trials in the placebo literature.

Which raises the question: Are there times when expectations run high and health outcomes low? Indeed, there are. This phenomenon, for example, was demonstrated experimentally in a study by Zunhammer and colleagues in which the route of placebo administration was changed from a patch to a pill for two groups: group 1, having initially had a negative or neutral response to the placebo patch; and group 2, having initially had a positive response to the placebo patch (60). Interestingly, changing the route of administration to a pill increased expectancy in the negative or neutral group and decreased expectancy in the positive group. Yet, despite their elevated expectancy, the treatment effect in the negative group, when compared with the positive group, was reduced: demonstrating the “lack of correspondence between treatment expectations and treatment responses.”(ibid.)

And so, as we see, the expectancy theory breaks down under close examination - especially at the level of the individual.

#### Belief and mindset

Another popular theory maintains that beliefs or mindsets determine placebo/nocebo effects. We place these two terms, belief and mindset, together as they are often used interchangeably (13). While many of the experimental outcomes in this realm are indeed valuable, the explanations themselves are often somewhat vague and confusing.

For example, Crum defines mindsets as follows: “A mindset is a setting of the mind; it’s a lens or a frame of mind through which we view the world to simplify the infinite number of potential interpretations at any given moment.” She adds, “mindset is a lens or frame of mind which orients an individual to a particular set of associations and expectancies.” (61) Other popular definitions are: “mindsets are lenses or frames of mind that guide individuals toward a set of expectations….,” and, “mindsets are similar to beliefs, as they steer motivation and attention….,” and “mindsets represent a simplified and stereotypical picture of one’s reality.” (13) A lens, a frame, a setting of mind, a simplified and stereotypical picture of one’s reality – not really the sharp terms that constitute a scientific explanation. More importantly, as we shall see, the studies that resort to these vague conceptions are all capable of a more concise and universal explanation.

Consider, for example, the study by Crum and Langer in which they randomly assigned hotel workers to either the information group or the control group (62). The information group was informed verbally and in writing of the “benefits of exercise and were informed that their daily housekeeping work satisfied the CDC’s recommendations for an active lifestyle.”(ibid.) Unfortunately, the study does not recite exactly what the subjects were told regarding the benefits of exercise; but the CDC states, “Regular physical activity can also lower your blood pressure and improve your cholesterol levels,” and “Being physically active can improve your brain health, help manage weight, reduce the risk of disease, strengthen bones and muscles, and improve your ability to do everyday activities.” (63) In other words, the information group was offered an idea, the essence of which was: If you do your daily work, then you will experience the health benefits specified by the Surgeon General. The communication of that one idea, which structurally is what Hammond calls a contingent suggestion, was the sole intervention (64).

Remarkably, “after only 4 weeks of knowing that their work is good exercise, the subjects in the informed group lost an average of 2 pounds, lowered their systolic BP by 10 points, and were significantly healthier as measured by body-fat percentage, BMI, and WHR.” Moreover, at the study’s conclusion, the informed group reported doing an average of 20% more daily exercise and double the previously reported amount of regular exercise – this despite no real increase in exercise within the group. In other words, their “mindset” about the nature of their work, exercise and health had changed. Accordingly, Crum and colleagues concluded that the experimental findings are attributable to “… a shift in mind-set initiated by the information given to them in the intervention.”

Why not simply state that the reported changes are due to the information given in the intervention and, perhaps, the circumstances that obtained during that communication (i.e. source, relationship)? Again, we see the same logical error as with expectancy theory. The authors acknowledge the initial element in the causal sequence producing the placebo effect is the communication of specific information; and yet, they attribute the study outcomes to a secondary effect – namely, a change in the participants’ mindsets.

#### Social context

The “social context” in which placebo-engendering ideas are conveyed has recently been the subject of much scrutiny (13, 22, 65, 66). Five categories of contextual factors have been identified: a) features of the healthcare worker, (b) features of the patient, (c) features of the therapeutic relationship, (d) features of the treatment, and (e) features of the healthcare setting (13). Presently, there is no single organizing principle under which these factors, and their role in generating placebo effects, can be understood. Nevertheless, many of the studies in this realm have brought important elements of placebo/nocebo phenomena to light.

For example, Howe randomly assigned participants to receive either positive or negative suggestions by a physician who demonstrated either high or low warmth and/or competence. Specifically, the positive group was told: “I am going to apply a hydrocortisone cream to the area where I pricked your skin [with histamine]. This cream is going to stop the reaction, which means it’s going to reduce itching and irritation.” The negative group was told, “I am going to apply a histamine agonist cream to the area where I pricked your skin. This cream is going to enhance the reaction, which means it’s going to increase the itching and irritation.” (22) Compared with a neutral group – no cream given following a histamine skin prick – the positive group had a smaller reaction, and the negative group had a larger reaction. (Perhaps owing to sample size, these differences were not statistically significant.) Moreover, among the positive group exposed to the “both high” condition - i.e., a physician with high competence and warmth – the reaction to histamine was significantly smaller relative to the average (p = 0.001). Howe concludes: “Positive expectations, when delivered by a warm and competent provider, diminished participants’ allergic responses. However, when delivered by a provider that was less warm and less competent, neither positive nor negative expectations had influence.”

Certainly, there is something of importance in these results. But as we have already discussed, caregivers do not deliver expectations; they deliver information. Expectations may arise in the patient secondarily, or they may not. What this study shows is that the relationship between the caregiver and patient somehow causes the information to actualize when the caregiver is perceived as “warm and competent,” but not when the caregiver is perceived as impersonal and incompetent. Any explanation of placebo/nocebo phenomena must somehow account for findings of this nature – findings that were evident even to the earliest researchers in the field (3, 8, 9).

#### The meaning model

In 2000, Brody introduced the Meaning Model to explain certain aspects of placebo phenomena (6). His view was: “An encounter with a healer is most likely to produce a positive placebo response when it changes the meaning of the illness experience for that individual in a positive direction.”(ibid.) Such a change in meaning, Brody asserts, is achieved by the caregiver in three ways: communicating a meaningful explanation of the illness, expressing care and concern, and imparting a sense of mastery and control to the patient.

Brody cites Egbert’s classic study as evidence in support of the meaning model (67). In that study, the night before surgery, randomly assigned pre-operative patients received either (a) a visit by the anesthesiologist in which postoperative pain was not discussed, or (b) a visit by the anesthesiologist in which the likelihood of postoperative pain was discussed along with its causes, what analgesics were available and how to ask for them, and what they themselves could do to reduce the pain. In other words, the sole intervention in this study was the unhurried communication of specific information to patients, the night before major surgery, by the person who would soon be assuming responsibility for their vital functions. No “props” – meaning, no sugar pills, no saline injections, no sham procedures. As Egbert reported, in the “special-care” group, post-operative narcotic use was reduced by half and patients were discharged home an average of 2 1/7 days sooner than patients in the control group.(ibid.) Again, for Brody, Egbert’s study constitutes proof of the meaning model. The special behavior of the anesthesiologist positively changed the meaning of the illness experience, and thereby produced a positive placebo effect.

But how did the simple communication of an idea result in faster healing and less discomfort? We are really no closer to an understanding of placebo with Brody’s meaning model than without it. Besides, one could argue with equal cogency that the meaning of greatest importance is not “the meaning of the illness experience,” but rather the meaning of the anesthesiologist’s communication – which, again, takes the abstract form of a contingent suggestion delivered by an authority-figure: specifically, “if you do the following post-operative activities, then you will heal faster and more comfortably.”

The term “meaning” came into different usage in 2002 through the work of Moerman and Jonas (68). These authors maintained that to consider placebo a thing, “flies in the face of the obvious.” Rather, they argue that it is the meaning of the intervention and its subsequent physiologic or psychological effects that eventuate in a “meaning response” - a term they prefer to placebo response. In other words, Moerman and Jonas recognize that what early-on had been called the “symbolic import” of the intervention (9) – namely, its “meaning” – is a prime determinant of placebo and nocebo effects.

In support of their thesis, they cite studies in which the color, number and brand of pills affected the clinical outcomes in accordance with the presumed meaning of those various properties (69, 70). However, as discussed earlier, it is important to distinguish conditioned responses from placebo/nocebo responses; and it is the authors’ conviction (and, we assume, most branding and marketing agencies as well) that, in these particular studies, conditioned responses primarily account for the results.

Nevertheless, Moerman and Jonas go on to cite the work of Desharnais in which the randomly assigned experimental group was informed their exercise program was designed to enhance psychological well-being, whereas the control group was given the same exercise program, but with no such information (71). While both groups improved their exercise tolerance, only the experimental group experienced enhanced “self-esteem.” Here we see a purer form of what Moerman and Jonas wish to convey. The meaning of the investigators’ message somehow actualized in the participants’ self-conception.

But how? Moerman and Jonas are clearly correct in their assessment of what constitutes a placebo/nocebo. As we have already established, placebo/nocebo is not a thing; it is a noetic intervention conveying meaning. Beyond that, we are beginning to discern that the meaning in question is most likely communicated via some form of contingent suggestion, implied or expressed, by an authority-figure. Isn’t it, then, fair to ask: What drives the perceived meaning of the communication to actualize in the patient’s/participant’s mind and/or body?

Unfortunately, none of the foregoing theories gets us any closer to answering this critical question.

## A better explanation

### The law of identification

As in the physical sciences, the answer lies in unseen – albeit, calculable - abstractions beyond the immediate events under study. For Newton, those unseen abstractions were mass and force; for Faraday and Maxwell, electromagnetic fields; for Einstein, space-time and energy. As we proceed to the new Law of Identification, we should bear in mind the words of Richard Feynman: “Science is only good if tells you about some experiment that has not been done….It is necessary to extend the ideas beyond where they have been tested.” (72) Our new law, therefore, should both subsume the now-extensive descriptions of placebo and nocebo phenomena and point the way to unexplored events and experiments.

We begin with the field of hypnosis. At first, one might wonder: What does hypnosis have to do with modern medicine and placebo effects? This perplexity stems from the centuries old conflation of hypnosis with trance. However, contemporary understandings of hypnosis have dispelled this tangled notion and now understand hypnosis to be a method by which trance and numerous other physical and psychological responses are induced. In other words, trance is an effect – hypnotic method is the cause. So, what is hypnotic method? It is a refinement of everyday communication that increases the likelihood a communicated idea will evoke, in the recipient of the communication, a psychological and/or physical response (73, 74). As such, hypnotic method resides at the far end of the communication continuum, often enabling its practitioners to persuade body and mind (75–79).

For centuries, the first idea communicated by persons using hypnotic method was that of trance (“Sleep!” commanded Dr. Bernheim), and so, trance ensued (80). Once trance was induced, all subsequent responses were mistakenly attributed to trance and not to the communicator or his/her relationship with the recipient. We now understand that the hypnotic influence of the operator is not a consequence of trance; if it were, how could one explain the operator’s ability to shift the subject from a normal waking state to a hyper-compliant trance state? (74) Instead, we understand hypnotic influence to be the compounded effect of four patterns which, once established, tend to persist: (a) Identification, (b) Rapport (an imitative pattern defined in measurable terms), (c) Linkage (whereby the operator’s words equal the subject’s experience) and (d) Conditioning. While each of these distinct patterns is significant, and while each can operate in concert with the others or separately, it is Identification that primarily accounts for placebo effects.

So, what is Identification?

Identification is an instinctual pattern that activates whenever one feels imperiled. It is the pattern whereby a helpless and dependent individual identifies an “authority figure” – vesting that authority figure with influence, whereby the ideas of the authority figure are unconsciously incorporated into the mind/body of the subject.

Identification begins in infancy. The human infant is born helpless and dependent, without support and guidance it cannot survive. Therefore, our species is hardwired to recognize and bond with a protective and succoring individual, one capable of ensuring survival. How does the helpless and dependent infant select its protector? As we have discussed elsewhere, the infant scans for any expression or micro-expression in the potential protector that incites uncertainty as to their ability to provide and protect.(ibid. pg.41-46) Whoever provokes the least uncertainty, the least doubt, is the person with whom the infant Identifies. Once that unconscious determination is made – that is, once identification occurs - information flows from the authority figure to the child: and the thoughts, ideas and actions of the one autonomously become the thoughts, ideas and actions of the other. In other words, as the human infant Identifies with its protector (usually, a parent) the reality of that protector becomes its own reality. As Dr. Bernie Seigel wrote: “As parents, we are, in a sense, our children’s first hypnotists….” (81)

This state of affairs is expressed in the following equation:


I= HD/U

Where I stands for Identification between the subject and another individual; H is the subject’s sense of helplessness; D is the subject’s sense of dependency; and U is the uncertainty the subject feels with regard to the other. (Note: We will discuss measurement of these factors shortly.)

This is the Law of Identification. Hence, the degree of identification between the subject and the other individual is (a) proportional to the product of the subject’s sense of helplessness and dependency, and (b) inversely proportional to the uncertainty the subject feels toward the other individual with regard to their care. The greater the helplessness and dependency, the stronger the identification. The greater the uncertainty in the other individual, the weaker the identification.

It should be emphasized that information acquired by a subject through identification with an authority figure enters at a level unaffected by reason and resistance, where it becomes “operative” – meaning, the information influences the subject’s actions and behaviors regardless whether the subject becomes conscious of it or not. Thus, in childhood, the words of the parent translate into the experience of the child: “That’s dangerous,” and the child senses a threat; “that’s yummy,” and the child experiences a pleasant flavor; while through other channels, the deeper ideas of the parent – i.e., religious, ethical, tribal, aesthetic, etc. – also become the operative ideas of the child, long before abstract thought and reason are even possible.

Later in life, the words of other authority figures may similarly define the identifier’s experience. Consider a woman, for example, in the throes of childbirth – helpless to diminish her pain, dependent on the epidural for relief, and feeling no uncertainty toward the anesthetist who is performing the procedure. Clearly, in this instance, the Law of Identification obtains. Therefore, it follows that when the authority figure conveys the idea of a “big bee sting” the patient feels more pain from the injection of local anesthetic than when the authority figure suggests numbness and comfort (82). Likewise, for surgical patients receiving an intravenous cannula: they are helpless to insert the cannula themselves, dependent on the clinician who (we are left to assume) incites little to no doubt about their ability to perform the procedure. Once again, the Law of Identification obtains. Therefore, it should not surprise us to discover that when the inserter communicates the idea “it’s a sharp scratch and it may sting a little,” patients report experiencing pain. Whereas, when the same inserter states that the tourniquet “allows the drip to be placed more comfortably,” patients do not report experiencing pain (83).

In fact, over the last two centuries, most of the positive clinical outcomes resulting from hypnotic interventions are best understood as the Law of Identification impelling ideas to actualization (75–80). Beyond that, the mysteries of cult behavior and Stockholm syndrome are also explained by this same Law, as the identifiers take on the reality of their cult leaders or captors.

But now the question is: How does the Law of Identification account for placebo and, possibly, nocebo effects?

To answer this question, we need first to recognize the universal notion expressed or implied in virtually every therapeutic encounter ever performed - including, placebo controlled trials. This idea is the Generic Placebo Instruction (GPI). In the abstract, it takes the form of the contingent suggestion (or, hypothetical proposition): “If you do X (i.e., the intervention), then Y (i.e., the prescribed outcomes) will occur.”

Again, X is the intervention. In order for the GPI to actualize, X must be:

- Credible at the outset. (Often this entails cultural and contextual factors, as well as conditioning.)
- Somewhat difficult to achieve, but achievable. (It cannot be so easy as to lose credibility.)
- Credible upon completion.

In other words, if X is not credible before, during and after the therapeutic encounter, uncertainty (U) regarding the authority-figure arises, and Identification (= HD/U) breaks down. And without identification, there is no force driving the GPI to actualization.

As to Y, we should note there is no requirement the prescribed outcomes be good or bad, desirable or undesirable. Often, they are both – that is, therapeutic effects and adverse side effects. Nevertheless, identification with the authority figure drives the prescribed outcomes to actualize in the subject, regardless of their subsequent appraisal. Thus, the same primordial *mechanism* drives both placebo and nocebo effects. Both are prescribed outcomes driven to actualize by the Law of Identification.

Of course, if Y is not specified, it will not be driven by the Law of Identification to occur. For example, if the potential benefits of a placebo are not presented, one cannot expect them to be induced. Blease and colleagues discuss this common omission, noting that out of 45 RCTs only one included a patient information leaflet that referenced potential beneficial effects from placebo (12). Clearly, this biases RCTs in favor of the experimental or active intervention (12). But the obverse is also true: namely, that potential adverse effects will not be induced when they are not communicated. This is demonstrated in Silvestri’s classic experiment, wherein patients receiving a beta-blocker were either (a) completely uninformed as to possible erectile dysfunction (ED) side effects, (b) allowed to discover on their own that ED was a possible side effect, or (c) informed by the researcher (i.e., the authority figure) that ED might occur. In other words, only the last group received the full GPI from an authority-figure in connection with the administration of the drug. Consequently, respective occurrences of ED in each group were (a) 3.1%, (b) 15,6% and (c) 31.2% (P<0.01): where group (a) represented the intrinsic incidence of ED caused by beta-blockers; group (b) revealed the effect of the patients’ own knowledge about possible side effects; and group (c) revealed the driving impact of the Law of Identification and the GPI in producing a nocebo effect (18).

To summarize: *Placebo/nocebo* is a noetic intervention that takes the form (implied or expressed) of the Generic Placebo Instruction and is delivered by someone with whom the subject has identified. As Turner asserted, “… a placebo effect does not require a [material] placebo.” (30) Nevertheless, props – like, pills, procedures or injections – may or may not accompany the intervention (X) to increase its credibility and, thereby, diminish uncertainty (U) in the clinician or researcher. Of course, the *meaning* of the GPI will depend on its content (X and Y) and on the recipient’s prior conditioning experiences (including, language learnings) and contextual factors. This perceived meaning is then driven to actualize by the Law of Identification - the “fundamental mechanism” Beecher long ago surmised must be at play (3). When subject-operator identification is strong, the prescribed outcomes (Y) – i.e., placebo and nocebo effects - will actualize. When subject-operator identification is weak, the prescribed outcomes, being mere words, will have no impact.

Just as we think of the *mechanism* behind falling objects as the Law of Gravity, so we should think of the *mechanism* behind placebo and nocebo effects as the Law of Identification.

## Implications of the new theory

Is our new theory a better explanation of placebo/nocebo phenomena? If it is, it must have greater reach, less variability and a predictive power that leads to the expansion of our observations, understandings and inquiries.

### Reach

Again, placebo is a noetic intervention in the form of the Generic Placebo Instruction (explicit or implicit), delivered by an authority figure with whom the subject has identified. It should be clear, this new theory meets all of our previously identified requirements. Moreover, provided conditioning events are categorized in a class of their own, as they should be, there are virtually no placebo interventions that do not fall under this definition. We see, for example, how even material placebo interventions – from sugar pills to sham surgeries – carry with them the GPI. (“If you take this pill/undergo this surgery, then you will….”) Beyond material placebo interventions, the GPI inheres in the wide range non-material interventions as well. For instance, under our present formulation *informed consent* is a noetic intervention identical in its elements to the placebo intervention. Specifically, it is information delivered by an authority figure to a patient who feels relatively helpless and dependent; and that information takes the form of a contingent suggestion – namely, “If you undergo this procedure (X), then (Y) the following risks and benefits may occur.”

This new definition of placebo also illuminates a serious error that has crept into the experimental design of numerous clinical trials: specifically, so-called “no-contact” and “wait-list” control groups (84–86). Both such “control groups” experience no placebo intervention whatsoever: there is no communication of the GPI from an authority figure to the study participants. Therefore, as observed by Turner and colleagues in 1994, no-contact and wait-list groups at best represent natural history controls; they do not represent placebo controls, whose fundamental purpose is to “control for” whatever placebo effects might manifest in their corresponding experimental groups (30). For, ideally (though, not always in practice) in randomized controlled trials, the same authority-figure delivers the same information (i.e., the GPI) to both experimental and placebo control groups; and it is this activity that produces – in accordance with the Law of Identification – placebo and nocebo effects in both groups.

We should also note that this definition of placebo frees us from the previous misconception that assumed deceit was somehow an essential element of placebos. The Generic Placebo Instruction entails no deceit whatsoever. True, this spurious notion had been largely dispelled by the proven efficacy of open-label placebos (OLP), which have been shown to work in many instances, except where direct contact with the clinician is not established (46, 87–89). However, with the disappearance of deceit, these same OLP studies elevated the question, “How, then, does placebo work?” If not deceit, then what?

Kaptchuk observed that many participants in OLP studies “… described the intervention as ‘crazy’ and overwhelmingly denied initial positive expectations during their intake and exit interviews.” (90) So, expectation (which we have already dismissed) cannot account for OLPs’ efficacy. But our new explanation can. Even in the first published OLP study (1965), researchers openly expressed the GPI to the open-label placebo cohort: “Many people,” they said, “with your kind of condition have also been helped by what are sometimes called ‘sugar pills,’ and we feel that a so-called sugar pill may help you, too…. I think this pill will help you as it has helped so many others.” (87) In other words, “If you (X) take this sugar pill, then (Y) you may be helped as have many others.” More recent OLP studies continue this explicit suggestion, as follows: “it’s an inert pill without physiological effects … it could help you, let’s see what happens.” (90) Again, “If (X) you take this inert pill, then (Y) it could help you.”

Our new theory, then, dispels the mystery surrounding open-label trials by observing within them the Generic Placebo Instruction, delivered by an individual with whom a relatively helpless and dependent patient or participant has identified.

But the reach of this new theory goes even further. One has only to realize that the same factors relating to subject, source, relationship and information (i.e., the GPI) that operate in placebo-controlled clinical trials also exist in virtually all therapeutic encounters. These are the common elements Beecher and Brody intuited and Welch adduced in his descriptions of trans-cultural healing rituals (3, 9, 91). In other words, inherent in virtually all therapeutic encounters - from the ministrations of Egyptian priests, to the dream healings of the Asclepians, to medieval barbers, Amazonian shamans, faith healers, and today’s physicians and surgeons – is the Generic Placebo Instruction, delivered by the one deemed least uncertain (and, therefore, most trustworthy/competent), and driven to actualization by the Law of Identification.

Thus, our new understanding demonstrates unrivaled reach, far beyond today’s popular theories. It encompasses not only the wide array of placebo/nocebo phenomena - including informed consent effects and OLP trials - but also hypnotic therapies, cult behavior, Stockholm syndrome and the vast array of therapeutic rituals practiced in various and sundry cultures around the world. And, our new theory shines light on the fallacy of designating no-contact and wait-list groups as placebo-control groups.

### Variability

The Law of Identification and GPI also function as organizing principles through which the wide array of factors affecting placebo/nocebo can be more clearly understood. For example, so-called contextual factors, encompassing literally dozens of variables (22, 67, 92–94), have been identified as somehow contributing to placebo effects through “…a cascade of psycho-neuro-immuno-endocrinological events capable of influencing patients’ nervous systems at multiple levels to release neurotransmitters and activate brain areas.” (13, pg278-9) But research on these factors has so far been largely descriptive.

Our new explanation, however, focuses on three essential factors that ultimately determine whether information (e.g., the GPI), when delivered by an authority-figure, will actualize. Those three factors all appertain to the patient/participant; they are (a) the person’s sense of helplessness, (b) their sense of dependency, and (c) their level of uncertainty as to the competence/trustability of the practitioner/researcher. All three factors are, of course, subject to the influence of the many variables already discovered by placebo science. Contextual factors, prior learnings, social factors and conditioning are all seen as elements contributing to the patient’s sense of helplessness, dependency or uncertainty regarding their caregiver. But ultimately, it is three factors that define the nature of the relationship between the source and the subject: specifically, whether identification occurs or not. If there is identification, the meaning the patient ascribes to the GPI will be driven to actualize by whatever psychobiological sequences are necessary to produce (Y) the prescribed outcomes.

Even the requirement that X, the intervention, be credible before, during and after completion bears ultimately on the patient’s/participant’s uncertainty regarding the authority-figure. For example, assume a “doctor’s” prescribed treatment is to have his patient stand on one foot and twirl a knotted sock with chicken livers in it over his head under a full moon. Depending on the cultural milieu, verbal and non-verbal cues, and other factors, this recommended treatment will either engender within the patient serious uncertainty as to the doctor’s trustworthiness, or it will inspire conviction that a cure is at hand. In other words, the credibility of the intervention will affect the patient’s assessment of the prescriber.

The question, then, becomes how to measure these three essential factors with precision and reliability, preferably through the use of uniform, validated tools. Fortunately, some early work in this direction has already begun. For example, psychometric tools for the measurement of helpless and dependency have been developed and, to a degree, validated (95, 96). And, the issue of uncertainty – specifically as it relates to “physician trustability” – has been preliminarily explored (97). However, in order to advance placebo science, other more refined measures of the three essential variables (e.g., HD/U) will need to be proffered and perfected. We propose such calibration tools in **Appendices 1**–**3**. In the end, our understandings will stand strongest when the concerted efforts of placebo researchers are all measured with reliability and precision on the same scales.

We should note that there is one additional factor at play in placebo-controlled trials – a factor not present in everyday clinical practice. Specifically, it is the question whether or not X (i.e., the intervention) is being administered. Recall, the Generic Placebo Instruction takes the form: “If X (the intervention), then Y (the prescribed outcomes).” In RCTs, where there is no absolute certainty as to whether X is being administered, the issue of belief arises. (Note: This is the one and only circumstance in which our new formulation admits of “belief” as a determinative factor in placebo phenomena.) If the patient believes they are receiving X - due to non-verbal hints, taste and color of the “prop,” mode of administration, side effects, or other cues – then the degree of identification with the caregiver will dictate the outcome. On the other hand, if the patient does not believe they are taking X, then the antecedent condition of the GPI (i.e., “If X…,”) is not met and no placebo effect should ensue. As Turner and colleagues noted, in RCTs “… physician and patient know there is a sham treatment and a real treatment, and outcomes are influenced by their expectancies and beliefs about which treatment the patient received. If either or both can guess (e.g., by side effects) which treatment the patient received, or if one treatment is more credible, this may bias the study results.” (30)

We see evidence of this in the work of Rutherford and Faria, where both teams demonstrate that drug-specific effects of antidepressants are not elicited if participants do not believe they are receiving the drug (59, 98). For example, Faria’s team found that when patients do not believe they are receiving the active agent, “the effect of the SSRI escitalopram … is reduced to a level that is not clinically meaningful.”(13.,pg. 217) This effect is further supported by the observation that the therapeutic response rate is lower in trials where the likelihood of receiving the inert agent is highest. Hence, placebo-participants in a two-arm trial (50% likelihood of receiving the inert agent) experience fewer therapeutic responses than placebo-participants in three- or four-arm trials (33% and 25% likelihood, respectively) (99).

Therefore, understanding and predicting placebo effects in blinded clinical trials requires, in addition to the three factors (e.g., HD/U) comprising the Law of Identification, an accurate and reliable measure of the subject’s belief as to whether or not they are receiving X, the intervention (**Appendix 4**).

In sum, our new explanation contains only 3 + 1 essential elements - all presumably capable of accurate and reliable measurement: namely, the subject’s (a) sense of helplessness, (b) dependency and (c) uncertainty with respect to the competence of the healthcare practitioner; plus, in RCTs, the subject’s level of belief that they are receiving X, the intervention. When these essential elements are known, and the components and meaning of the GPI (both X and Y) are understood, placebo and nocebo effects become both comprehensible and predictable.

### Predictive power

Does our new explanation predict outcomes hitherto unforeseen? Indeed, it does.

Once standardized, measurement of the three essential factors in the Law of Identification will yield previously undiscoverable information. To date, virtually all placebo/nocebo studies have relied on data aggregation within cohorts for statistical comparison of averages: predictability at the level of the individual has been absent (13, 30, 100). However, using reliable, validated measures, researchers will be able to calculate the level of identification (I = HD/U) for each and every patient/participant; and so, a “threshold of actualization” may be discovered, above which all or most participants will actualize the prescribed outcomes (Y); and if not such a threshold, then, a probability table correlating the probability of actualization with various strata of identification. Thus, future researchers, and perhaps even practicing clinicians, will have access to precision, personalized information capable of predicting who will and who will not be a placebo/nocebo responder.

Additionally, the Law of Identification indicates that whenever and wherever the subject feels most helpless and dependent, and has the least uncertainty as to the competence of the practitioner, then identification will be maximal. It is in those moments the words of the clinician, whether they follow the GPI format or take some other form, will have their greatest impact. Which raises the question: When and where do we find patients most helpless and dependent, and most trusting in their clinician?

Here are two examples. The first is when a patient presents *in extremis* to the Emergency Department and their outcome depends upon the astute functioning of their Emergency Physician. Here, there are already confirmatory reports of the Emergency Physician’s words evoking remarkable physical and psychological outcomes, including hemostasis, cardioversion, airway dilatation, non-manipulative joint reduction, anesthesia and analgesia (74, 101). The second example is in the moment just prior to induction of general anesthesia for a major surgical procedure, when the anesthesiologist is administering the pre-op sedative. Imagine if, in such a moment, instead of asking the patient to count backwards the anesthesiologist offers suggestions for hemostasis, post-operative comfort and rapid healing. We should expect – and there is, again, confirmatory (albeit, anecdotal) evidence to suggest this would happen - less blood loss, fewer analgesics used and faster healing in the group offered the GPI as compared with the count-backwards control group (67).

While these instances are presented as opportunities to enhance therapeutic outcomes by recognizing the intensity of the patient’s identification with the authority figure and offering positive suggestions, there are equivalent opportunities for deleterious effects should negative suggestions be offered. Again, as Hauser wrote: “Patients are highly receptive to negative suggestion, particularly in situations perceived as existentially threatening” – that is, in situations where the patient’s sense of helplessness and dependency is maximal (67). We see this perhaps most clearly when informed consent is delivered (9, 17, 102). For instance, a systematic review of 21 RCTs of tricyclic antidepressants (TCAs) revealed a higher rate of adverse events in these placebo cohorts than in the placebo cohorts of 122 selective serotonin reuptake inhibitor studies (SSRIs) - presumably, because informed consent disclosed fewer potential adverse side effects with SSRIs than with TCAs (103). Similarly, fewer adverse events and absenteeism resulted from influenza vaccines when vaccinees were informed of the proportion of patients who tolerated the procedure well, as compared with when they were informed of the proportion of patients who suffered unpleasant side effects (104).

But what if informed consent could be honestly presented, containing all the requisite information, and yet somehow assign positive possibilities to subjects without also assigning negative possibilities to them? Remember, the *meaning* of the GPI is always a result of the subject’s interpretation (conscious and unconscious); as such, it is capable of being influenced by the same linguistic niceties often employed in more subtle forms of communication. Accordingly, the informed consent GPI could take the form: “If you undergo this intervention X, then, Y = (a) on the one hand, *some people might* experience the following negative effects, while (b) on the other hand, people *perhaps like you experience* little or no side effects and the following benefits….” This formulation exploits the distinction linguists draw between general referential indices (e.g. some people, them, others, etc.) and specific referential indices (e.g. you, your, yourself) – a distinction often used in hypnosis to subtly direct suggestions (105). There are anecdotal data suggesting this method’s efficacy (74). But looking ahead, like all the aforementioned predictions arising from our new theory, this refinement of the informed consent procedure is eminently capable of controlled experimental confirmation or disconfirmation.

### Further clarifications

Understanding that *placebo effects* are consequences of the Law of Identification driving the GPI to actualization dispels the fog of numerous misconceptions.

Foremost is the notion that placebo effects are somehow undesirable intrusions into the realm of real medicine. For example, citing the work of Litten and colleagues, the authors of a recent chapter on placebo effects wrote that the presence of up to 93.5% placebo response rates “…makes it difficult to discern a quantifiable treatment effect for modestly effective investigational medications.” (13, 106) They go on to urge the identification of genetic variants (and other factors) associated with lower placebo response rates. While of some interest, perhaps, this largely misses the mark. For who needs a modestly effective medication when there already exists a 93.5% effective placebo? After all, our purpose as clinicians is to promote healing and sustain health. If, at times, that goal can be better achieved with placebo, presumably with fewer side effects than pharmacologic agents, then why not use placebo? The question should not be how to lower placebo responses, but rather as Jonas writes: “How do we as clinicians and clinician-scientists integrate placebos into practice.”(13, pg.11)

Another prominent misconception is that placebos may exert potential harm from “… unintended violation of [patient] autonomy through unappreciated power imbalances favoring the provider and/or undetected undue influence by the provider.”(13, pg.28) For decades we have known of real harm (i.e., nocebo effects) being done by both active agents and placebos; but because we have not understood the cause of these effects, we have not been able to mitigate them. We now better understand that, indeed, there does exist a previously undetected and underappreciated influence on the part of the provider. That influence is a function of our patients’ sense of helplessness and dependency and our relative lack of uncertainty with respect to the management of their health problems. At times, we may be able to diminish identification by reducing our patients’ sense of helplessness and dependency, thereby returning to our patients their sense of agency. Or, we may raise their uncertainty (and thereby reduce identification) by simply saying “I don’t know” when asked about their future or other unknowables. At other times, for whatever reasons, we may find ourselves unable to reduce our patients’ identification. In such cases, we are now at least cognizant of our influence and of our patients’ susceptibilities; and so, we are more likely to speak carefully and with deliberation. Thus, inadvertent harm from careless speech – so common in today’s clinical practice – can now be avoided, and in its place, positive effects from honest and salutary suggestions can be achieved.

Finally, there is this – the overriding misconception of reductionists: namely, with the discovery of the physical mechanism underlying placebo comes understanding. This is not true. Physical intermediates – including, endorphins, neurotransmitters, peptides, etc. – have been known for decades (13, 29). And yet, confusion regarding the world of placebo phenomena has persisted.

Our new theory teaches that the causal sequences leading to placebo and nocebo effects begin with a noetic event (i.e. the transmission of information), and only after such an event occurs do physical sequences follow. This should surprise no one. You intend to raise your hand, and up it goes. First the intention, then the nerve impulses, then the motor end-plate activation, and finally the chemistry of muscular contraction and relaxation. This is a learned response, subject to the Law of Association. All the known physical events involved in hand raising represent, if you will, the interior science of voluntary movement. Similarly, all of the physical events intermediating placebo effects represent the interior science of placebo phenomena. And, interior science is abundantly useful. However, it is at the frontier that the overarching understandings of science reside; for it is there that one discovers the laws governing observable events. With respect to placebo and nocebo effects, we believe our new theory now offers us that overarching understanding.

## Discussion

In the 16^th^ century, Paracelsus observed: “Man has received from Nature both the destroyer of health and the preserver of health.” (107) Our survey of placebo science has revealed the actions of both the destroyer (i.e. nocebo effects) and the preserver (i.e. placebo effects). We have come to understand that each of these innate capacities can be activated by the delivery of information from an individual to a recipient, provided the recipient has identified with that individual; for it is through identification that the meaning of the message – influenced as it is by such factors as culture, context and conditioning – reaches the unconscious, where it actualizes through psychological or physical sequences known and unknown (54). As the celebrated cardiologist and Nobel laureate, Bernard Lown, M.D., wrote: “Words are the most powerful tool a doctor possesses, but words...can maim as well as heal.” (27).

Hence, our new theory compels us to realize that unlike elsewhere in our lives - where we may lack expertise, and so, induce little if any identification – our communications as professionals can have profound impacts on the health and healing of others. Our words matter. Our gestures and expressions matter: from greetings and goodbyes, to informed consent, to what we say and how we say it when offering a medication or performing a procedure, to our often-tenuous prognostications. And so, as Hauser suggests, “skill in conveying positive suggestions and avoiding negative ones should receive more attention,” (27) especially, as Mommaerts writes, our skills in “communication with the subconscious” which should now reside at “the center of medicine.” (54) And where better to learn the requisite communication methods and nuances of *noetic medicine* than from the very discipline that provided us with our understanding of the Law of Identification – namely, clinical hypnosis (74, 105, 108–112).

Certainly there is work to be done: validating tools to reliably calibrate the prime determinants of identification, designing experiments to test our theory (including those mentioned above), conducting and replicating those experiments. But the potential consequences of these efforts – especially, their translational effects on clinical medicine – offer sound reason for the work ahead.

## Data Availability

The original contributions presented in the study are included in the article/**Supplementary Material**. Further inquiries can be directed to the corresponding author.
