# Supplementary material for: Placebo and the law of identification
Source: Front Psychiatry. 2024 Dec 6;15:1474558. doi: 10.3389/fpsyt.2024.1474558 (PMC11659211; doi:10.3389/fpsyt.2024.1474558)
Supplement: Supplementary file 1 [file DataSheet1.pdf]

Appendix 1

IDENTIFICATION/DEPENDENCY INDEX

The following questions relate specifically to your health circumstances: namely, the circumstances that have brought you to this questionnaire. Please answer each question only as it relates to your present health circumstances and NOT to other aspects of your life.

Please give every answer a numerical score from 1-5, in accordance with the following scale:

1-Disagree
2-Somewhat disagree
3-Not sure
4. Somewhat agree
5. Agree

QUESTIONS	SCORE
1. In my current circumstances, I need to rely on an expert for help.	____
2. With respect to current circumstances, I cannot achieve my goal alone.	____
3. I am relying on my healthcare provider/researcher to provide whatever is needed to achieve my current health goal.	____
4. I don't mind depending on an expert in the current situation.	____
5. In fact, I am or will be depending on an expert to help me in the current circumstances.	____

Appendix 2

IDENTIFICATION/HELPLESSNESS INDEX

The following questions relate specifically to your health circumstances: namely, the circumstances that have brought you to this questionnaire. Please answer each question only as it relates to your present health circumstances and NOT to other aspects of your life.

Please give every answer a numerical score from 1-5, in accordance with the following scale:

1-Disagree
2-Somewhat disagree
3-Not sure
4. Somewhat agree
5. Agree

QUESTIONS	SCORE
1. I need help achieving my health goal.	____
2. I have no idea how to achieve my health goal by myself.	____
3. In fact, I do not believe I can achieve my health goal by myself.	____
4. There is little or nothing I alone can do to achieve my health goal.	____
5. I have no more control over my health issue(s) than I do over the weather.	____

____

Appendix 3

IDENTIFICATION/UNCERTAINTY INDEX

The following questions relate specifically to your health circumstances: namely, the circumstances that have brought you to this questionnaire. Please answer each question only as it relates to your present health circumstances and NOT to other aspects of your life.

Please give every answer a numerical score from 1-5, in accordance with the following scale:

1-Disagree
2-Somewhat disagree
3-Not sure
4. Somewhat agree
5. Agree

QUESTIONS	SCORE
1. I have my doubts about my healthcare provider's ability to help me.	____
2. I wish my faith in my healthcare practitioner were stronger.	____
3. I'm not at all sure my healthcare provider can help to fix my current problem.	____
4. If I had a more confidence in my healthcare provider, I think I could have more success with my health issue(s).	____
5. I suspect a different healthcare provider could be more effective in helping me with my current issue(s).	____

Appendix 4

IDENTIFICATION/BELIEF IN TAKING ACTIVE AGENT INDEX

The following questions relate specifically to the placebo-controlled study you are participating in. Please answer each question only as it relates to the current study and NOT to any other aspect of your life.

Please give every answer a numerical score from 1-5, in accordance with the following scale:

1-Disagree
2-Somewhat disagree
3-Not sure
4. Somewhat agree
5. Agree

QUESTIONS	SCORE
1. I believe I am taking the active agent in this study.	____
2. I can tell with certainty I am not taking the placebo.	____
3. There are certain cues that convince me I am taking the active agent and NOT the placebo.	____
4. What I am taking simply cannot be the placebo.	____
5. If this were the placebo I am taking, I wouldn't be having the responses I am having.	____
6. Only the active agent in this study could be having the effect on me that I am experiencing.	____
